# Relationship of tobacco smoking and smoking-related DNA methylation with epigenetic age acceleration

**DOI:** 10.18632/oncotarget.9795

**Published:** 2016-06-02

**Authors:** Xu Gao, Yan Zhang, Lutz Philipp Breitling, Hermann Brenner

**Affiliations:** ^1^ Division of Clinical Epidemiology and Aging Research, German Cancer Research Center (DKFZ), Heidelberg, Germany; ^2^ Division of Preventive Oncology, German Cancer Research Center (DKFZ) and National Center for Tumor Diseases (NCT), Heidelberg, Germany; ^3^ German Cancer Consortium (DKTK), German Cancer Research Center (DKFZ), Heidelberg, Germany; ^4^ Pneumology and Respiratory Critical Care Medicine, Thoraxklinik, University of Heidelberg, Heidelberg, Germany

**Keywords:** tobacco smoking, epigenetic clock, age acceleration, AHRR, whole blood sample, Gerotarget

## Abstract

Recent studies have identified biomarkers of chronological age based on DNA methylation levels. Since active smoking contributes to a wide spectrum of aging-related diseases in adults, this study intended to examine whether active smoking exposure could accelerate the DNA methylation age in forms of age acceleration (AA, residuals of the DNA methylation age estimate regressed on chronological age). We obtained the DNA methylation profiles in whole blood samples by Illumina Infinium Human Methylation450 Beadchip array in two independent subsamples of the ESTHER study and calculated their DNA methylation ages by two recently proposed algorithms. None of the self-reported smoking indicators (smoking status, cumulative exposure and smoking cessation time) or serum cotinine levels was significantly associated with AA. On the contrary, we successfully confirmed that 66 out of 150 smoking-related CpG sites were associated with AA, even after correction for multiple testing (FDR <0.05). We further built a smoking index (SI) based on these loci and demonstrated a monotonic dose-response relationship of this index with AA. In conclusion, DNA methylation-based biological indicators for current and past smoking exposure, but not self-reported smoking information or serum cotinine levels, were found to be related to DNA methylation defined AA. Further research should address potential mechanisms underlying the observed patterns, such as potential reflections of susceptibility to environmental hazards in both smoking related methylation changes and methylation defined AA.

## INTRODUCTION

Tobacco smoking is a major public health problem, associated with substantial preventable morbidity globally [1]. In particular, active smoking in adults accounts for a large proportion of age-related diseases, including various forms of cancer, respiratory and cardiovascular diseases [2]. Recent studies have demonstrated a role of DNA methylation, one of the main forms of epigenetic modification, in the pathways of smoking and smoking-induced diseases *via* regulating gene expression and genome stability [3]. An increasing number of smoking related CpG sites in various genes, such as *AHRR*, *F2RL3* and *GPR15*, have been discovered by epigenome-wide association studies (EWASs) based on whole blood samples, and have been shown to be useful as quantitive biomarkers of current and past smoking exposure and predictors of smoking-associated health risks [4, 5]. Recently, Teschendorff et al. constructed a smoking index based on 1501 smoking-related loci and showed that smoking-related methylation indices could be useful risk indicators of smoking-induced health disorders [6].

Recent studies have also disclosed age-related alterations of DNA methylation [7], and an “epigenetic clock” for DNA methylation age based on known age-related biomarkers has been shown to predict an individual's chronological age with high accuracy [8]. Horvath and Hannum et al. developed two broadly accepted measurements for determining DNA methylation age in multiple tissues and blood samples, respectively [9, 10]. The discrepancy between methylation age and chronological age (defined as age acceleration, denoted AA) was found to be heritable and has been suggested to be applied as an index of disproportionate aging. A positive AA indicates that an individual is ahead of his or her chronological age, and a negative one suggests an individual is biologically “younger” than reflected by the chronological age [7, 9]. Follow-up investigations linked AA to lifestyle factors, environmental hazards, as well as stressful life events, and further revealed that AA was a biologically meaningful biomarker associated with aging-related diseases [11–21].

Given the association of smoking with multiple age-related diseases [2], it would appear plausible that smoking may have an impact on AA. However, the few studies assessing this relationship have reported conflicting findings. Horvath et al. and Marioni et al. did not find significant associations of self-reported smoking with DNA methylation age determined in peripheral blood samples [11, 14], while Beach et al. recently reported such an association for the most robust smoking-related locus, cg05575921 (*AHRR*), as a biomarker of smoking exposure [15]. To further explore a possible role of smoking in AA, we conducted a comprehensive analysis of the associations of self-reported smoking, serum cotinine levels (an established biomarker of current smoking exposure) and smoking-associated methylation signatures with AA in a large population-based study.

## RESULTS

### Participant characteristics

Characteristics of the study population in the discovery and validation panels were comparable with respect to chronological age, DNA methylation ages, smoking behaviors, as well as lifestyle factors, and are summarized in Table 1. Average age in the two subsets was about 62 years, and chronological ages were highly correlated with corresponding methylation ages (r ≥0.75, Figure S1). Hannum et al.'s methylation ages of both panels were higher than chronological ages and ages computed by Horvath's approach. More than half of the participants in each subset were ever smokers (current /former smokers), and around 18% still smoked at the time of recruitment. In both subsets, the proportion of men was much higher in current smokers than that in never smokers: 60.8% *vs*. 29.4% in the discovery panel and 48.0% *vs*. 21.1% in the validation panel (data not included in the table). Average cumulative smoking exposure in current smokers was considerably higher than that of former smokers in both panels. Average cessation time for former smokers in the two subsets was also similar, approximately 17 years. Cotinine levels of current smokers (64.1 ng/ml) were much higher than levels of never (4.1 ng/ml) and former (7.3 ng/ml) smokers in the discovery panel.

**Table 1:** Study population characteristics in discovery and validation panels ^a^

Characteristics	Discovery Panel	Validation Panel
**N**	978	531
**Age (years)**	62.1 (6.5)	62.0 (6.6)
**Methylation age 1 (Horvath, years)**	61.6 (7.1)	63.4 (7.5)
**Methylation age 2 (Hannum, years)**	68.7 (7.2)	67.3 (6.8)
**Sex (male)**	495 (50.6%)	207 (39.0%)
**Smoking status**		
Current smoker	181 (18.5%)	98 (18.4%)
Former smoker	328 (33.5%)	182 (34.3%)
Never smoker	469 (48.0%)	251 (47.3%)
**Pack-years of smoking**		
Current smokers	36.8 (19.3)	33.9 (17.5)
Former smokers	23.3 (16.3)	19.9 (15.1)
**Smoking cessation time (years) ^b^**	17.3 (11.3)	17.6 (10.6)
**Serum cotinine levels (ng/ml)^c^**		
Current smoker	64.1 (29.2)	NA
Former smoker	7.3 (19.5)	NA
Never smoker	4.1 (14.0)	NA
**Body mass index (kg/m^2^)^d^**		
Underweight or normal weight (<25.0)	245 (25.1%)	162 (30.5%)
Overweight (25.0-<30.0)	472 (48.4%)	228 (42.9%)
Obese (≥30.0 )	258 (26.5%)	141 (26.6%)
**Alcohol consumption^e^**		
Abstainer	311 (34.1%)	169 (34.4%)
Low	531 (58.2%)	290 (59.1%)
Intermediate	53 (5.8%)	27 (5.5%)
High	17 (1.9%)	5 (1.0%)
**Physical activity^f^**		
Inactive	189 (19.3%)	109 (20.5%)
Low	433 (44.3%)	261 (49.2%)
Medium or high	356 (36.4%)	161 (30.3%)

a Mean values (SD) for continuous variables and n (%) for categorical variables;

b Former smokers only, data missing for 9 and 3 participants, respectively, in discovery and validation panels; cessation time equals age at recruitment minus age at cessation;

c Only measured in the discovery panel, not applicable (NA) in validation panel;

d Data missing for 3 participants in discovery panel;

e Data missing for 66 and 40 participants, respectively, in discovery and validation panels. Categories defined as follows: abstainer, low [women: 0 -<20 g/d, men: 0 -<40 g/d], intermediate [20 -<40 g/d and 40 -<60 g/d, respectively], high [≥40 g/d and ≥60 g/d, respectively];

f Categories defined as follows: inactive [ < 1h of physical activity/week], medium or high [≥2 h of vigorous and ≥ 2 h of light physical activity/week], low [other];

### Associations between smoking indicators and age accelerations

In the analyses of associations of self-reported measures of smoking and serum cotinine levels with AA, two linear regression models were employed (details are presented in Methods), controlling for potential confounding factors. None of the self-reported smoking indicators (smoking status, cumulative exposure and smoking cessation time) or serum cotinine levels was significantly associated with AA in the discovery panel (Table 2, Figure S2). Furthermore, we selected a total of 150 loci related to active smoking, which were identified ≥2 times in previous smoking EWASs, as biomarkers of smoking exposure [4], excluding one locus (cg11314684) which was part of Horvath's predictor of methylation age [9]. Associations between AA according to Horvath's and Hannum et al.'s algorithms (dependent variable) and methylation levels of these candidates (independent variable) were assessed by two mixed linear regression models (Models 1, 2) with methylation assay batch as random effect and increasing adjustment for potential confounders (details are presented in Methods). However, even after fully controlling for confounding factors (Model 2), 103 and 94 of the 150 CpG candidates passed the threshold of FDR < 0.05 and thus demonstrated significant associations with AA according to the Horvath's and Hannum et al.'s algorithms in the discovery phase, respectively (Figure S3). Subsequently, we selected 83 AA-related loci based on both algorithms and then verified them in the validation samples (Table S1, Figure S3, FDR < 0.05). 74 and 70 of these loci were confirmed as significantly related loci for AA derived according to Horvath's and Hannum et al.'s algorithms by the fully-adjusted model, respectively. Eventually, a total of 66 smoking-related CpG sites that were statistically significant in both algorithms (Table S1, Figure S3, FDR < 0.05). We additionally conducted a sensitivity analysis in the validation panel adjusting for covariates of Model 2 plus the prevalence of cardiovascular diseases (yes/no), diabetes (yes/no) and cancer (yes/no). In this sensitivity analysis, associations remained statistically significant for all of the 66 loci with similar results (data not shown). The 66 CpG sites were eventually designated as the loci associated with DNA methylation aging in whole blood samples. Four hypermethylated smoking-related loci in smokers also showed positive correlations with AA (Table S1). Among the remaining negatively correlated CpG sites, 12 loci had Spearman's coefficients less than or equal to −0.20 for both AA algorithms (Table 3). They are located at seven genes: *2q37.1* (*n* = 1), *AHRR* (*n* = 3), *AVPR1B* (*n* = 1), *HUS1* (*n* = 1), *KCNQ1* (*n* = 2), *NCRNA00114* (*n* = 1), *NFE2* (*n* = 1) and two unnamed genomic regions. Among these, methylation differentials in the locus cg07123182 (*KCNQ1*) were associated with the largest alterations in AA in regression analyses for both AA algorithms.

**Table 2:** Associations of self-reported smoking indicators and cotinine levels with age acceleration in the discovery panel

Self-reported smoking indicators			Age acceleration (Horvath)			Age acceleration (Hannum)		
			Estimate	SE	*p*-value	Estimate	SE	*p*-value
**Model 1^a^**	**Smoking status**	Current smoker	−0.17	0.44	0.70	−0.37	0.41	0.37
		Former smoker	0.18	0.38	0.64	−0.46	0.36	0.20
		Never smoker	Ref			Ref		
	**Cumulative smoking (pack-year) ^b^**		3.9 e-3	0.013	0.76	7.3 e-3	0.012	0.55
	**Smoking cessation time (years) ^c^**		4.2 e-3	0.023	0.85	0.026	0.023	0.26
	**Serum cotinine levels (ng/ml)**		−1.6 e-3	5.3 e-3	0.76	−3.7 e-3	5.0 e-3	0.46
**Model 2^a^**	**Smoking status**	Current smoker	−0.13	0.46	0.88	−0.34	0.43	0.43
		Former smoker	0.10	0.39	0.79	−0.51	0.36	0.16
		Never smoker	Ref			Ref		
	**Cumulative smoking (pack-year)^b^**		2.0 e-3	0.012	0.87	9.9 e-3	0.012	0.39
	**Smoking cessation time (years)^c^**		2.2 e-3	0.024	0.93	0.020	0.024	0.39
	**Serum cotinine levels (ng/ml)**		1.1 e-3	5.6 e-3	0.85	−1.3 e-3	5.2 e-3	0.80

a Model 1: Adjusted for age (years) and gender; Model 2: Adjusted for age (years), gender, alcohol consumption (abstainer/ low/ intermediate/ high), body mass index (BMI, underweight or normal weight/ overweight/ obese), physical activity (inactive/ low/ medium or high), the prevalence of cardiovascular diseases (yes/no), diabetes (yes/no) and cancer (yes/no).;

b A pack-year was defined as having smoked 20 cigarettes per day for 1 year, including current and former smokers from discovery panel;

c Cessation time defined as age at the time of recruitment minus age at cessation, only including former smokers from discovery panel;

**Table 3:** Top 12 significantly age acceleration related CpG sites in validation panel ^a^

CpG sites	Genes	Mean (SD)^b^	Age acceleration (Horvath)				Age acceleration (Hannum)			
			Correlation coefficients^c^	Effect size (SE)^d^	*p*-value	FDR	Correlation coefficients^c^	Effect size (SE)^d^	*p*-value	FDR
cg03329539	*2q37.1*	0.38 (0.036)	−0.23	−23.9 (6.0)	8.7 e-5	2.0 e-4	−0.27	−16.6 (4.5)	2.8 e-4	6.0 e-4
cg01899089	*AHRR*	0.49 (0.033)	−0.30	−41.0 (5.6)	1.8 e-12	7.3 e-11	−0.33	−29.8 (4.3)	9.9 e-12	1.6 e-10
cg05575921	*AHRR*	0.85 (0.041)	−0.21	−5.8 (2.1)	5.3 e-3	8.2 e-3	−0.25	−5.5 (1.5)	4.2 e-4	8.3 e-4
cg24090911	*AHRR*	0.73 (0.034)	−0.20	−28.9 (5.8)	7.8 e-7	3.1 e-6	−0.33	−31.2 (4.2)	5.6 e-13	1.5 e-11
cg20295214	*AVPR1B*	0.76 (0.029)	−0.26	−31.9 (6.6)	1.6 e-6	6.2 e-6	−0.27	−24.9 (4.9)	6.0 e-7	3.3 e-6
cg10190813	*HUS1*	0.17 (0.026)	−0.25	−39.5 (9.5)	4.1 e-5	9.6 e-5	−0.23	−18.8 (7.4)	0.011	0.015
cg01744331	*KCNQ1*	0.90 (0.032)	−0.21	−35.2 (6.9)	4.8 e-7	2.0 e-6	−0.25	−25.3 (5.3)	2.5 e-6	1.1 e-5
cg07123182	*KCNQ1*	0.93 (0.019)	−0.20	−48.9 (8.7)	4.2 e-8	2.7 e-7	−0.25	−41.4 (6.5)	5.3 e-10	6.3 e-9
cg06595162	*NCRNA00114*	0.72 (0.026)	−0.24	−48.5 (6.8)	1.5 e-12	7.3 e-11	−0.25	−34.7 (5.1)	4.7 e-11	6.5 e-10
cg04158018	*NFE2*	0.30 (0.040)	−0.21	−24.5 (6.3)	1.1 e-4	2.3 e-4	−0.23	−20.8 (4.7)	9.4 e-6	3.3 e-5
cg25305703		0.65 (0.051)	−0.29	−23.6 (3.5)	4.1 e-11	8.5 e-10	−0.36	−21.6 (2.5)	2.8 e-16	1.2 e-14
cg16201146		0.67 (0.045)	−0.24	−37.9 (6.4)	7.4 e-9	6.2 e-8	−0.20	−41.5 (4.6)	9.7 e-18	8.1 e-16

a 12 loci with correlation coefficients ≤ −0.20;

b Data of never smokers;

c Spearman's Rank-Order Correlation coefficients;

d Adjusted for age (years), gender, random batch effects, leukocyte distribution (Houseman algorithm [21]), alcohol consumption (abstainer/ low/ intermediate/ high), body mass index (BMI, underweight or normal weight/ overweight/ obese) and physical activity (inactive/ low/ medium or high); The beta coefficients from regression models were reported as effect sizes;

### Smoking index (SI) and cg05575921 (*AHRR*)

**Figure 1:**
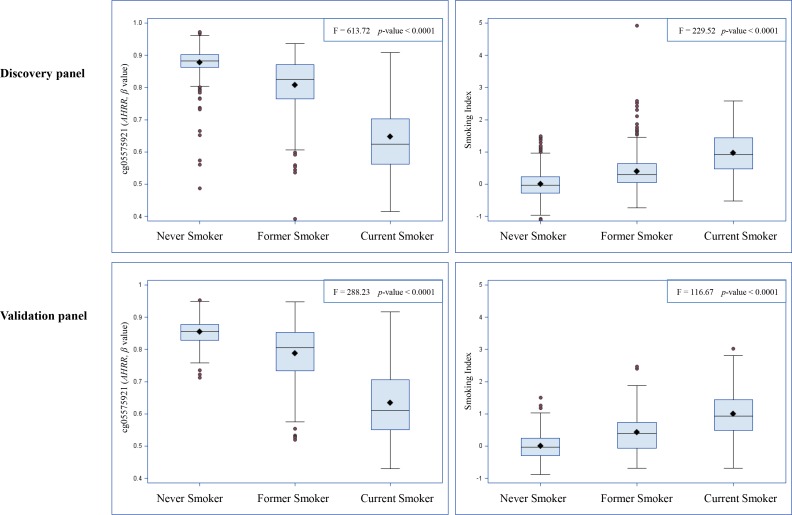
Distributions of cg05575921 and smoking index according to self-reported smoking status

**Figure 2:**
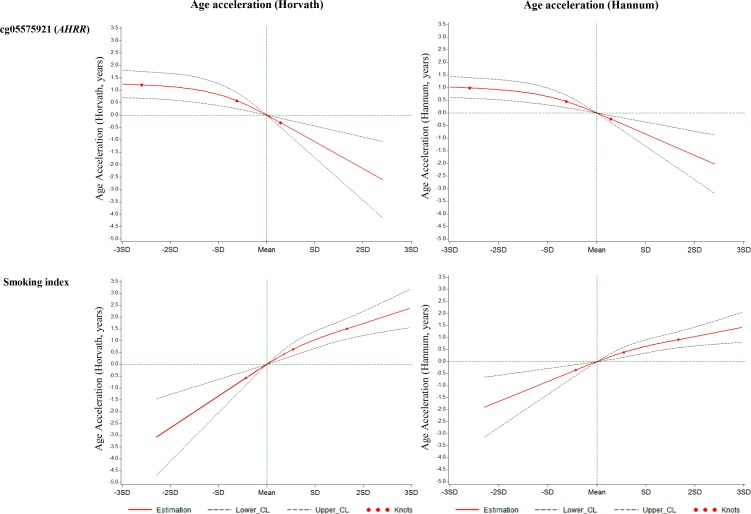
Graphs of the best-fitting models for the associations of cg05575921 and the smoking index with age accelerations in validation panel Red lines: Estimation; Dashed lines: Confidence limits; Red dots: Knots (25^th^, 50^th^ and 75^th^ quartiles); Green lines: reference lines.

We constructed a SI based on the 66 selected smoking-related loci and compared this indicator to one of the most robust smoking-related biomarkers cg05575921 (*AHRR*), which is known to be hypomethylated under smoking exposure, and the SI estimated based on 1501 loci identified in the study by Teschendorff et al. (Teschendorff SI) [6]. First, as shown in Figure 1, both cg05575921 and SI based on 66 loci were strongly associated with smoking status: levels in current smokers were lower (for cg05575921)/ higher (for SI) than those in never smokers and levels of former smokers were in the intermediate position. Furthermore, the results of mixed linear regression models showed that both methylation markers were significantly associated with both AA algorithms (Table 4). However, the Teschendorff SI was associated with AA according to Horvath's algorithm, but not with AA according to Hannum et al.'s algorithm (Table 4). Its correlations with both AA algorithms were much weaker than that of SI based on 66 loci (Table S2). In addition, the positive correlations of SI with the AA algorithms were stronger than the negative correlations between cg05575921 and the AA algorithms (Table S2). Another index based on 58 CpG sites without the eight *AHRR* loci further demonstrated similar correlations with AA and cg05575921 as SI (Table S2). The SI was also associated with the prevalence of cardiovascular diseases (*p* = 0.014, OR = 1.7 (95CI: 1.2 - 2.6, per unit of SI)), but not with the prevalence of diabetes (*p* = 0.19) or cancer (*p* = 0.39) in logistic regression models in the validation panel. Lastly, we explored the dose-response relationships of both smoking indicators with the AA algorithms. For both smoking indicators (Figures 2 and S4), monotonic associations with the AA algorithms were observed (monotonic decrease for cg05575921, monotonic increase for SI). An increase in the SI by one standard deviation was roughly associated an one-year increase in AA derived according to the Horvath's algorithm, and with a 0.5 −1 year increase in AA derived according to the Hannum et al.'s algorithm.

**Table 4:** Associations of age accelerations with epigenetic smoking indicators

Smoking indicators		Age acceleration (Horvath)				Age acceleration (Hannum)			
		Model 1^a^		Model 2^b^		Model 1		Model 2	
		Effect size (SE)^c^	*p*-value	Estimate (SE)	*p*-value	Effect size (SE)	*p*-value	Estimate (SE)	*p*-value
**Discovery panel**	cg05575921	−3.69 (1.43)	0.0102	−6.08 (1.43)	< 0.0001	−3.25 (1.36)	0.017	−3.54 (1.28)	0.006
	Smoking index	2.11 (0.26)	< 0.0001	2.15 (0.26)	< 0.0001	2.25 (0.24)	< 0.0001	1.80 (0.23)	< 0.0001
	Teschendorff SI	1.84 (0.72)	< 0.0001	2.56 (0.27)	< 0.0001	1.11 (0.68)	0.102	1.96 (1.05)	0.192
**Validation panel**	cg05575921	−4.21 (1.98)	0.034	−5.79 (2.07)	0.0053	−5.58 (1.53)	0.0003	−5.48 (1.54)	0.0004
	Smoking index	1.93 (0.33)	< 0.0001	2.27 (0.35)	< 0.0001	1.71 (0.26)	< 0.0001	1.65 (0.26)	< 0.0001
	Teschendorff SI	1.42 (0.58)	< 0.0001	2.00 (0.83)	0.0001	2.31 (1.28)	0.073	2.44 (1.41)	0.083

a Adjusted for age (years), sex and random batch effects;

b Adjusted for age (years), sex, random batch effects, leukocyte distribution (Houseman algorithm [41]), alcohol consumption (abstainer/ low/ intermediate/ high), body mass index (BMI, underweight or normal weight/ overweight/ obese) and physical activity (inactive/ low/ medium or high);

c The beta coefficients from regression models were reported as effect sizes;

## DISCUSSION

To our knowledge, this is the first systematic investigation exploring the association between active smoking exposure and its biological correlates with DNA methylation age in whole blood samples, based on two independent subgroups of a population-based cohort of older adults from Germany. None of the self-reported smoking indicators, including smoking status, cumulative exposure and time since smoking cessation, or serum cotinine levels was significantly associated with AA. However, we found 66 previously confirmed smoking-related CpG sites to be also associated with AA. A smoking index (SI) based on these loci and methylation at a robust mono-biomarker of active smoking cg05575921 (*AHRR*) showed monotonic associations with AA. An association with Horvath's algorithm of AA was also found for the Teschendorff SI.

Smoking has been considered as a critical factor in the risk of a number of age-related adverse health outcomes [2, 22, 23]. However, none of the genomic regions that become either hypermethylated or hypomethylated with aging has been identified in smoking EWASs [4, 7], even though the AA derived according to Hannum et al.'s algorithm is linked closely to one CpG site, cg05575921, which had been identified as an epigenetic indicator of smoking exposure in previous EWASs [15, 24, 25]. Our study confirmed this locus and additionally identified 65 loci that were associated with both smoking and AA as well. It appears plausible that these smoking-related loci might contribute to some of the aging-related health outcomes. In particular, eight out of the 66 loci were located at *AHRR*, a well-known tumor suppressor gene, which was suggested to be involved in or is involved in the metabolism of endogenous toxins from smoking [26]. We also identified another three smoking-related genomic regions with more than two AA-related sites that were associated with aging-related diseases: *AVPR1B* (Arginine Vasopressin Receptor 1B) contributes to overweight and might related with diabetes development [27], *CNTNAP2* (Contactin Associated Protein-Like 2) is demonstrated to be associated with several mental diseases (e.g. autism, schizophrenia, epilepsy and depression) [28–30], and *KCNQ1* (Voltage Gated KQT-Like Subfamily Q, Member 1) is another well-known gene for type 2 diabetes [31]. Additionally, the identified AA-related locus cg19713429 was located at *CAPZB* (Capping Protein Actin Filament Muscle Z-Line, Beta), which contains a locus cg13319175 that was used as an indicator in Horvath's algorithm [9]. No associations were found with other well-established smoking-related loci, like cg03636183 (*F2RL3*) and cg19859270 (*GPR15*) [4]. The strongest association with AA, in particular a strong monotonic dose-response relationship based on restrict cubic spline regression, was found for a smoking index encompassing all 66 smoking-related CpG sites.

Although our findings of a lack of association between self-reported measures of smoking and AA, along with robust associations between smoking-related methylation markers and AA appear to be inconsistent and hard to reconcile at first sight, there are multiple mechanisms that might explain the observed patterns. First, it is well known that susceptibility of individuals to adverse health effects of environmental hazards strongly varies between individuals [32, 33]. For example, despite the fact that smoking strongly increases the risk of multiple age-related diseases, some proportion of smokers (especially light smokers) stays relatively healthy up to old age [34], and the health risks associated with smoking may depend on a number of factors such as genetic polymorphisms in detoxifying enzymes or co-prevalence of other risk factors [35, 36]. It appears well conceivable that both smoking-related methylation markers as well as methylation defined AA might to some extent reflect increased susceptibility to environmental hazards such as smoking. Along the same lines, the possibility has to be kept in mind that smoking-related methylation changes may not only reflect smoking exposure, but also that similar methylation changes might be induced by other environmental hazards, such as alcohol consumption, nutritional or lifestyle factors [37, 38], or by potentially interactive or addictive effects between those factors and smoking, which may likewise be associated with increased risk of age-related diseases and age acceleration [7, 23]. Finally, self-reported smoking exposure is known to be subject to inaccuracies, e.g. by recall bias or willful underreporting [39]. Smoking-related methylation markers may more accurately reflect true smoking exposure and thereby facilitate disclosure of smoking-related adverse health effects. While our results of strong associations between smoking-related methylation markers and AA are intriguing, further research is needed to unravel the underlying mechanisms, such as those discussed above.

Major strengths of the present study include the relatively large sample size with detailed information on a broad range of covariates in a large population-based cohort and the comprehensive validation in an independent group, as well as the estimation of DNA methylation ages by two widely accepted methods. There are also several limitations that have to be considered in the interpretation of our study. Associations of smoking with DNA methylation in whole blood might be influenced by smoking-induced shifts in leukocyte distribution [40]. In order to remove potential confounding by this factor, our analyses adjusted for leukocyte distribution by the Houseman algorithm [41]. Stressful life events, another potential determinant of epigenetic aging [17, 18], could not be controlled for as information on this potential confounder was not collected in our study. In addition, our study was undertaken in an almost exclusively Caucasian population and results may not be generalized to other populations. For instance, different smoking associated CpG sites have been identified in Asian and African populations [42–44]. Hence, additional studies in other ethnic groups are required to get a more comprehensive picture of the potential role of smoking and smoking-related DNA methylation in age acceleration. Finally, due to the lack of potential genetic predictors of SI or mQTLs for smoking-related loci, we were not able to disentangle causal pathways *via* Mendelian Randomisation-type approaches which should be followed in further research [45, 46].

Along with the modernization of human society, expanding environmental hazards, beyond conventional factors like smoking and alcohol consumption, i.e. emerging factors like novel chemicals, biohazards and diseases, may be accelerating our biological aging in silence [7]. As the reliability of self-reported or externally measured exposure to such hazards remains limited, measurement of biologically relevant internal doses in epigenetic assays might be a promising approach for establishing related health hazards [47], and monitoring DNA methylation age may provide a window to target early interventions in high-risk individuals. Beyond advancing the understanding of AA and its association with active smoking, our study highlights the potential of surrogate epigenetic indicators, such as the smoking index and DNA methylation age, to quantify biologically relevant exposures and health outcomes. Further research should explore whether and to what extent such epigenetic signatures can be of value in clinical practice to enhance risk stratification and evaluation of preventive and therapeutic interventions.

## MATERIALS AND METHODS

### Study population

Study subjects were selected from the ESTHER study, an ongoing statewide population-based cohort study conducted in Saarland, a state located in southwest Germany. Details of the study design have been reported previously [48]. Briefly, 9949 older adults (aged 50-75 years) were enrolled by their general practitioners during a routine health check-up between July 2000 and December 2002, and followed up thereafter. Two independent subgroups were selected as discovery panel and validation panel, respectively, for epigenetic analyses. The discovery panel included 1000 participants recruited consecutively at the start of ESTHER study between July and October 2000. The validation panel included 548 participants randomly selected from participants recruited between October 2000 and March 2001. The study was approved by the ethics committees of the University of Heidelberg and the state medical board of Saarland, Germany. Written informed consent was obtained from all participants.

### Data collection

Information on socio-demographic characteristics, lifestyle factors and health status at baseline was obtained by standardized self-administered questionnaires. Participants were asked about past and present cigarette, cigar and pipe smoking behaviors and were then categorized into current, former and never smokers. Detailed information on smoking history was also obtained from questionnaires, including age at initiation and smoking intensities at various ages, as well as age of quitting smoking for former smokers. 22 and 17 participants were excluded from the discovery and the validation panel, respectively, due to missing information on smoking status. Additional information on body mass index (BMI) was extracted from a standardized form filled by the general practitioners during the health check-ups. Blood samples were taken during the health check-up and stored at −80°C until further processing. DNA from whole blood samples was extracted using a salting out procedure [49].

### Laboratory data

DNA methylation profiles were assessed by the Illumina Infinium Human Methylation 450 Beadchip array (Illumina, San Diego, CA, USA). As previously described [50], samples were analyzed following the manufacturer's instruction at the Genomics and Proteomics Core Facility of the German Cancer Research Center, Heidelberg, Germany. Illumina'sGenomeStudio^®^ (version 2011.1; Illumina.Inc.) was employed to extract DNA methylation signals from the scanned arrays (Module version 1.9.0; Illumina.Inc.). The methylation status of a specific CpG site was quantified as a β value ranging from 0 (no methylation) to 1 (full methylation). According to the manufacturer's protocol, no background correction was done and data were normalized to internal controls provided by the manufacturer. All controls were checked for inconsistencies in each measured plate. Signals of probes with a detection *p*-value > 0.05 were excluded from analysis. We used the Illumina normalization and preprocessing method implemented in Illumina's Genomestudio (“Illumina normalization”). In addition, as previously described [51], we measured the cotinine levels in serum samples of the discovery panel, using the customized version of an enzyme-linked immunosorbent assay (Inspec II-Cotinine-EIA; Mahsan Diagnostika).

### DNA methylation age

DNA methylation age of each participant was calculated by two algorithms proposed by Horvath [9] and Hannum et al. [10]. Horvath's algorithm, which was derived from a range of tissues and cell types, uses 353 probes targeted in the Illumina 27k and 450k methylation arrays. Methylation ages of study participants according to Horvath's algorithm were estimated by online calculator (http://labs.genetics.ucla.edu/horvath/dnamage/), where background-corrected beta values were pre-processed using the calculator's internal normalization method [9]. Hannum's algorithm is based on 71 methylation probes from the Illumina 450k methylation array which were derived as the best age predictors with data generated from whole blood DNA [10]. Hannum's methylation age was determined as the sum of the methylation beta values multiplied by the reported effect sizes of the predictors. Age accelerations (AAs) were determined as discrepancies between methylation and chronological age in the form of residuals, which have a mean of 0 and thus represent positive and negative deviations from chronological age in years. The residuals were calculated by a linear regression procedure in which methylation age was the outcome and chronological age was the independent variable.

### Statistical analyses

Study populations in the discovery and validation panels were described with respect to major socio-demographic characteristics, DNA methylation age, lifestyle factors, smoking behavior and serum cotinine levels.

Initially, we investigated the associations of self-reported smoking indicators (smoking status [current/ former/ never smoker], cumulative smoking exposure [pack-years, in current and former smokers] and smoking cessation time [years, in former smokers only], independent variables) and cotinine levels (ng/ml, independent variable) with AA (dependent variable) derived according to both algorithms (Horvath & Hannum et al.) in the discovery panel. Two linear regression models were employed, controlling for potential confounding factors. Model 1 was adjusted for age (years) and sex, and Model 2 was additionally adjusted for alcohol consumption (abstainer, low [women: 0 - < 20 g/d, men: 0 - < 40 g/d], intermediate [20 - < 40 g/d and 40 - < 60 g/d, respectively], high [≥40 g/d and ≥60 g/d, respectively]), body mass index (BMI, kg/m^2^, underweight or normal weight [ < 25], overweight [25 - < 30], obese [≥30]), physical activity (inactive [ < 1h of physical activity/week], medium or high [≥2 h of vigorous and ≥2 h of light physical activity/week], low [other]), the prevalence of cardiovascular diseases (yes/no), diabetes (yes/no) and cancer (yes/no). Indicators with a *p*-value < 0.05 were considered as AA-associated factors.

Furthermore, we selected a total of 150 loci related to active smoking, which were identified ≥2 times in previous smoking EWASs, as biomarkers of smoking exposure [4], excluding one locus (cg11314684) which was part of Horvath's predictor of methylation age [9]. Associations of their methylation levels (independent variables) with AA (dependent variable) were analyzed by two mixed linear regression models with methylation assay batch as random effect, controlling for potential confounding factors in both panels. Model 1 was adjusted for age (years) and sex. Model 2 was additionally adjusted for the leukocyte distribution estimated by the Houseman algorithm [41], alcohol consumption, body mass index and physical activity. After correction for multiple testing by the false discovery rate (FDR, Benjamini-Hochberg method [52]), CpG sites with corrected *p*-values < 0.05 were selected from the discovery panel and then replicated in the validation panel. Loci with FDR < 0.05 in the validation panel were eventually considered as AA-associated loci. We additionally conducted a sensitivity analysis in the validation panel adjusting for covariates of Model 2 plus the prevalence of cardiovascular diseases (yes/no), diabetes (yes/no) and cancer (yes/no) to confirm the identified AA-associated loci.

Finally, we used the identified AA associated loci to construct a smoking index (SI) according to Teschendorff et al.'s algorithm [6], to measure the deviation of DNA methylation in a given sample from a normal reference, with the mean taken over the identified loci. In more detail, we computed the mean β value (*μ_c_*) and standard deviation (*σ_c_*) across the never smokers of the given dataset, and then defined the SI as

$$\text{SI}\left(s\right)=\frac{1}{n}\sum_{c}^{n}W_{c}\frac{\beta_{\text{cs}}-\mu_{c}}{\sigma_{c}}$$

where *W_c_* is +1(−1) if the smoking-associated CpG, *c*, is hypermethylated (hypomethylated) in smokers and where *β_c_* is the β value of this CpG in samples *s* [6]. We calculated the SI for each participant in both panels based on the validated AA associated loci, and then compared it with the single epigenetic smoking indicator cg05575921 (*AHRR*) used in the study by Beach et al., [15] and the SI estimated based on 1501 loci identified in the study by Teschendorff et al. (Teschendorff SI) [6]. Mutual correlations of these indicators and AA were assessed by Spearman's correlation coefficients, and the associations of the smoking indicators with AA were assessed by mixed linear regression (Models 1 and 2). The associations of SI with the prevalence of aging-related diseases (Yes/No), including cardiovascular diseases, diabetes and cancer, were analyzed by logistic regression with adjustment for potential covariates in the validation panel. Additionally, we employed restricted cubic spline functions using the SAS macro from Desquilbet et al. to evaluate the dose-response relationships of both indicators with AAs [53], controlling for age (years), sex, the leukocyte distribution estimated by Houseman's algorithm, alcohol consumption, body mass index and physical activity (categorical variables were transformed into dummy variables). The 25^th^, 50^th^ and 75^th^ percentiles of the SI were chosen as the knots. Data cleaning and all aforementioned analyses were performed by SAS version 9.3 (SAS Institute Inc., Cary, NC, USA).

## SUPPLEMENTARY MATERIAL






