# Supplementary material for: Relationship of tobacco smoking and smoking-related DNA methylation with epigenetic age acceleration
Source: Oncotarget. 2016 Jun 2;7(30):46878–89. doi: 10.18632/oncotarget.9795 (PMC5216910; doi:10.18632/oncotarget.9795)
Supplement: Supplementary file 1 [file oncotarget-07-46878-s001.pdf]

Relationship of tobacco smoking and smoking-related DNA methylation with epigenetic age acceleration

Supplementary Material

Table S1: Details of associations between smoking-related loci and age acceleration

This table includes 2 subsets: 1. Details of discovery panel (83 loci); 2. Details of validation panel (66 loci).

For Table S1, please see the attached Excel file

Table S2: Correlation coefficients between age acceleration and epigenetic smoking indicators (Spearman's Correlation) in discovery and validation panels.

For Table S2, please see the attached Excel file

Discovery panel

Validation panel

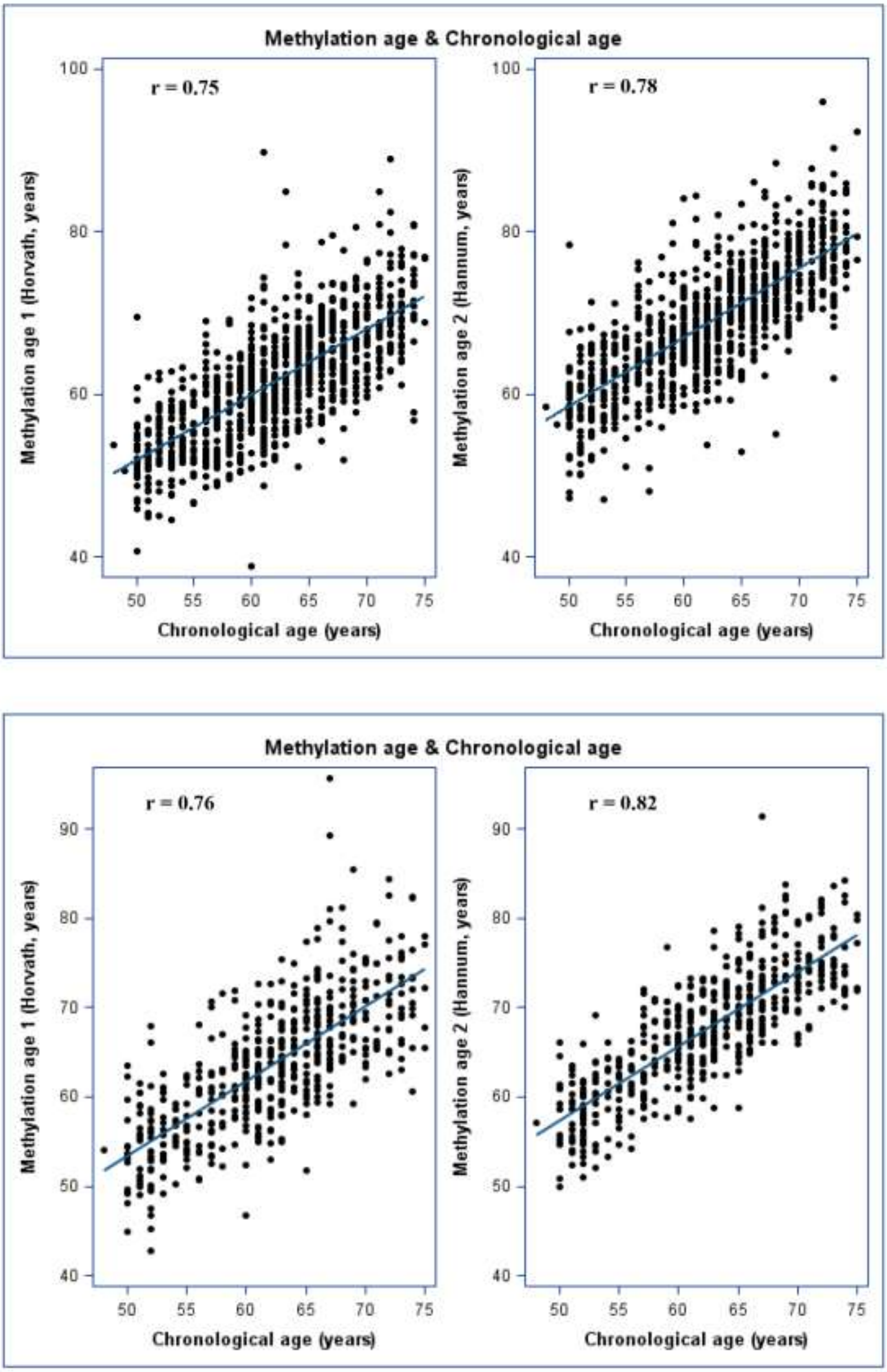

Figure S1: Plots of predicted methylation ages against chronological age.

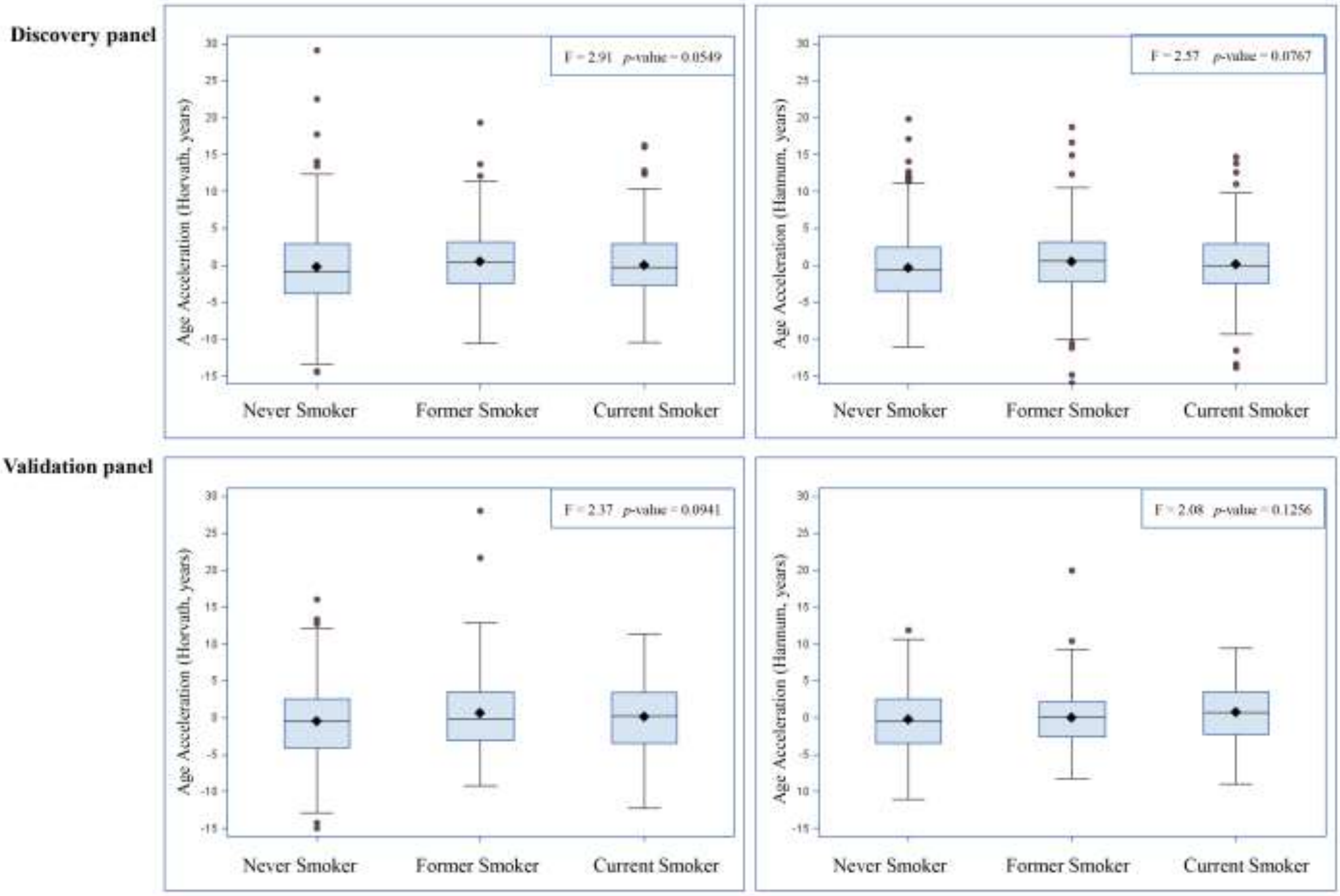

Figure S2: Distributions of age acceleration according to self-reported smoking in discovery and validation panels.

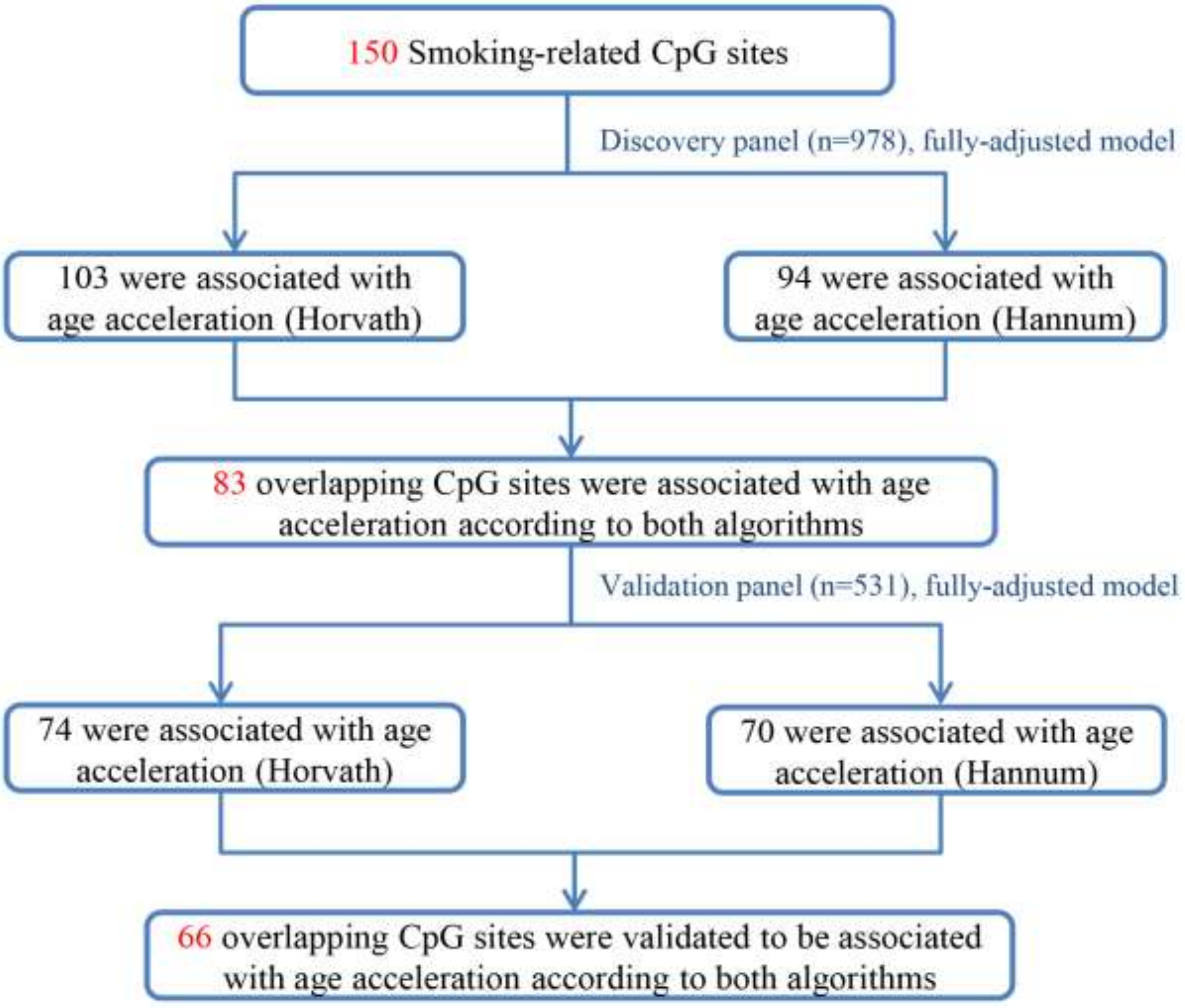

Figure S3: Flow chart of analyses between AA according to Horvath's and Hannum et al.'s algorithms and methylation levels of smoking-related CpG sites.

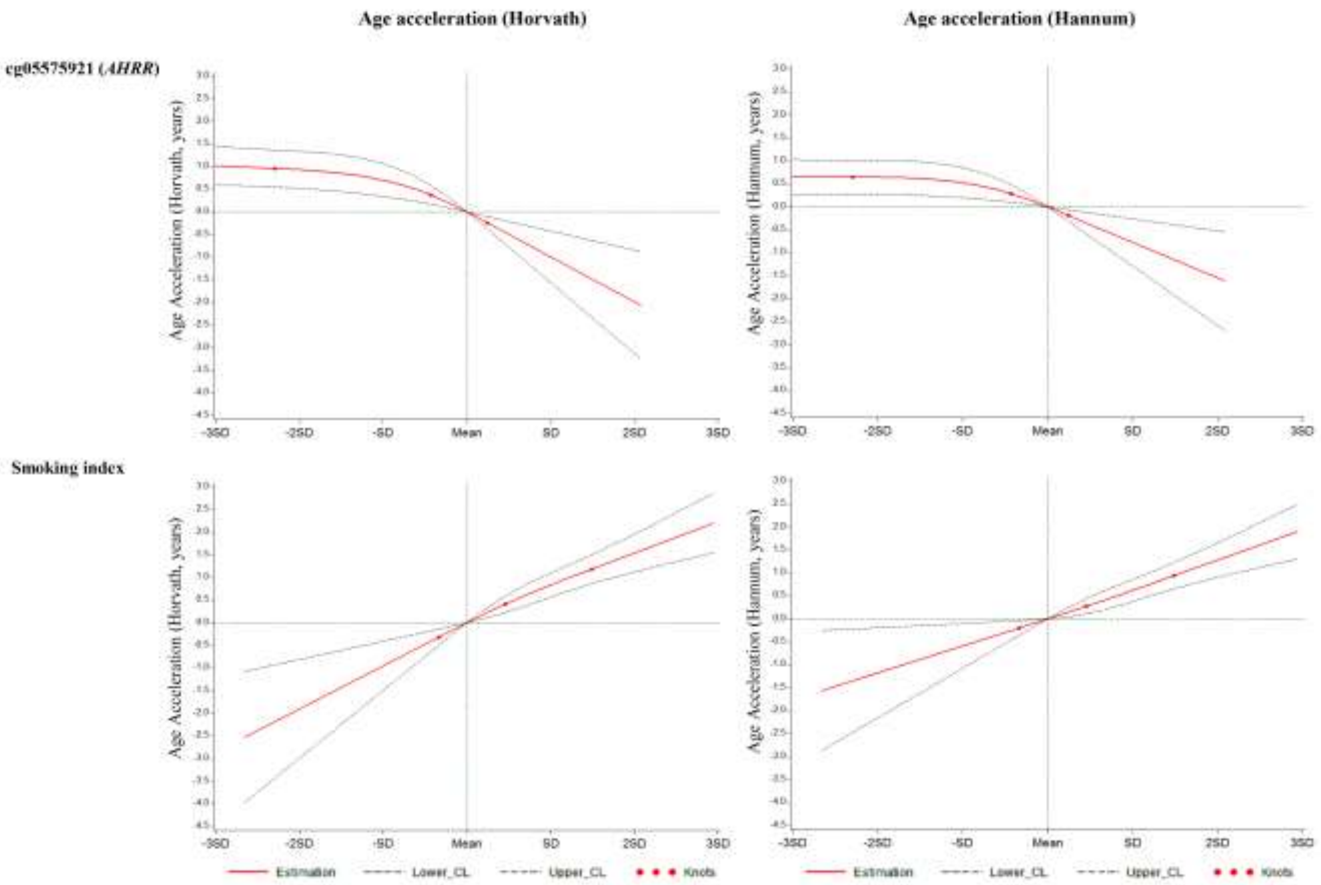

Figure S4: Graphs of the best-fitting models for the associations of cg05575921 and the smoking index with age accelerations in discovery panel.

Red lines: Estimation; Dashed lines: Confidence limits; Red dots: Knots (25th, 50th and 75th quartiles); Green lines: reference lines.
