# Supplementary material for: Relationship of tobacco smoking and smoking-related DNA methylation with epigenetic age acceleration
Source: Oncotarget. 2016 Jun 2;7(30):46878–89. doi: 10.18632/oncotarget.9795 (PMC5216910; doi:10.18632/oncotarget.9795)
Supplement: Supplementary file 3 [file oncotarget-07-46878-s003.pdf]

Table S2 Correlation coefficients between age acceleration and epigenetic smoking indicators (Spearman's Rank-Order Correlation) in discovery and validation panels [a]

	Variables	Age acceleration (Horvath)	Age acceleration (Hannum)	cg05575921	Smoking index	Smoking index without AHRR loci	Teschendorff SI
**Discovery**	**Age acceleration (Horvath)**	1	0.55834***	-0.17793***	0.27468***	0.26910***	0.15950**
	**Age acceleration (Hannum)**	0.55834***	1	-0.20149***	0.30729***	0.30793***	0.01489
	**cg05575921**	-0.17793***	-0.20149***	1	-0.77145***	-0.72599***	-0.18050***
	**Smoking index**	0.27468***	0.30729***	-0.77145***	1	0.99414***	0.26034***
	**Smoking index without AHRR loci**	0.26910***	0.30793***	-0.72599***	0.99414***	1	0.25981***
	**Teschendorff SI**	0.15950**	0.01489	-0.18050***	0.26034***	0.25981***	1
**Validation**	**Age acceleration (Horvath)**	1	0.53728***	-0.20557***	0.27411***	0.26816***	0.15027**
	**Age acceleration (Hannum)**	0.53728***	1	-0.24916***	0.28812***	0.27816***	0.01440
	**cg05575921**	-0.20557***	-0.24916***	1	-0.66987***	-0.61813***	-0.34503***
	**Smoking index**	0.27411***	0.28812***	-0.66987***	1	0.99393***	0.70362***
	**Smoking index without AHRR loci**	0.26816***	0.27816***	-0.61813***	0.99393***	1	0.71084***
	**Teschendorff SI**	0.15027**	0.01440	-0.34503***	0.70362***	0.71084***	1

a: Categories of *p*-values for the correlation coefficients: *** : $p<0.0001$; **: $p<0.01$; * : $p<0.05$; none: $p \geq 0.05$
